# Orexinergic descending inhibitory pathway mediates linalool odor-induced analgesia in mice

**DOI:** 10.1038/s41598-021-88359-5

**Published:** 2021-04-29

**Authors:** Yurina Higa, Hideki Kashiwadani, Mitsutaka Sugimura, Tomoyuki Kuwaki

**Affiliations:** 1 Department of Physiology, Graduate School of Medical and Dental Sciences, Kagoshima University, Kagoshima, 890-8544 Japan; 2 Department of Dental Anesthesiology, Graduate School of Medical and Dental Sciences, Kagoshima University, Kagoshima, 890-8544 Japan

**Keywords:** Neuroscience, Physiology

## Abstract

Linalool odor exposure induces an analgesic effect in mice. This effect disappeared in the anosmic model mice, indicating that olfactory input evoked by linalool odor triggered this effect. Furthermore, hypothalamic orexinergic neurons play a pivotal role in this effect. However, the neuronal circuit mechanisms underlying this effect have not been fully addressed. In this study, we focused on the descending orexinergic projection to the spinal cord and examined whether this pathway contributes to the effect. We assessed the effect of intrathecal administration of orexin receptor antagonists on linalool odor-induced analgesia in the tail capsaicin test. We found that the selective orexin type 1 receptor antagonist, but not the selective orexin type 2 receptor antagonist, prevented the odor-induced analgesic effect. Furthermore, immunohistochemical analyses of c-Fos expression induced by the capsaicin test revealed that neuronal activity of spinal cord neurons was suppressed by linalool odor exposure, which was prevented by intrathecal administration of the orexin 1 receptor antagonist. These results indicate that linalool odor exposure drives the orexinergic descending pathway and suppresses nociceptive information flow at the spinal level.

## Introduction

Orexins (also known as hypocretins) were first identified as neuropeptides regulating feeding behavior^1,2^. They originate from a common precursor polypeptide called prepro-orexin^1^. Orexins mediate their effects via two distinct G-protein coupled receptors (orexin type 1 receptor (OX1 receptor) and orexin type 2 receptor (OX2 receptor))^1^. The affinity of orexin-A is similar to that of both OX1 receptor and OX2 receptor, whereas orexin-B preferentially binds to OX2 receptor^1^. Although orexinergic neurons are highly localized in the perifornical area of the lateral hypothalamus, they virtually project their axons over the entire brain^1,3–5^.

After the initial discovery of the orexigenic function, several lines of evidence have revealed that orexins are also involved in other autonomic and physiological functions such as arousal^6,7^, cardiovascular^8–11^, respiratory^11,12^, neuroendocrine^13^, thermoregulatory^14,15^, and pain processing^16–20^. The analgesic (pain relief) effect of orexins was first reported by Bingham and his colleagues^21^. Intracerebroventricular administration of orexin peptide induced significant analgesic effects. Thereafter, microinjection of orexins or orexin receptor agonist revealed that orexin could regulate pain processing at several brain regions such as the locus coeruleus^22–24^, periaqueductal gray matter^19^, rostral ventromedial medulla^25^, ventral tegmental area^26^, and spinal cord^16,24,27–29^. Thus, orexinergic system could affect various level of pain processing.

Recently, we reported that odor exposure to linalool, one of the main odorous compounds in lavender extract, induced an analgesic effect in mice^20^. The effect disappeared in the anosmic model mice, indicating that olfactory input evoked by the linalool odor drives intrinsic analgesic circuits. Furthermore, neither orexin peptide-deficient mice nor selective ablation of orexin neurons in mice showed linalool odor-induced analgesia, indicating that the orexinergic system plays a pivotal role in the analgesic effect^20^. However, the orexinergic neuronal circuit mechanisms underlying odor-induced analgesia are largely unknown. In this study, we focused on the descending orexinergic projection to the spinal cord^24,30,31^ and hypothesized that linalool odor exposure drives the descending pathway and suppresses nociceptive information flow in the spinal cord.

## Results

### Five-minute exposure to linalool odor induced significant analgesic effects

**Figure 1:**
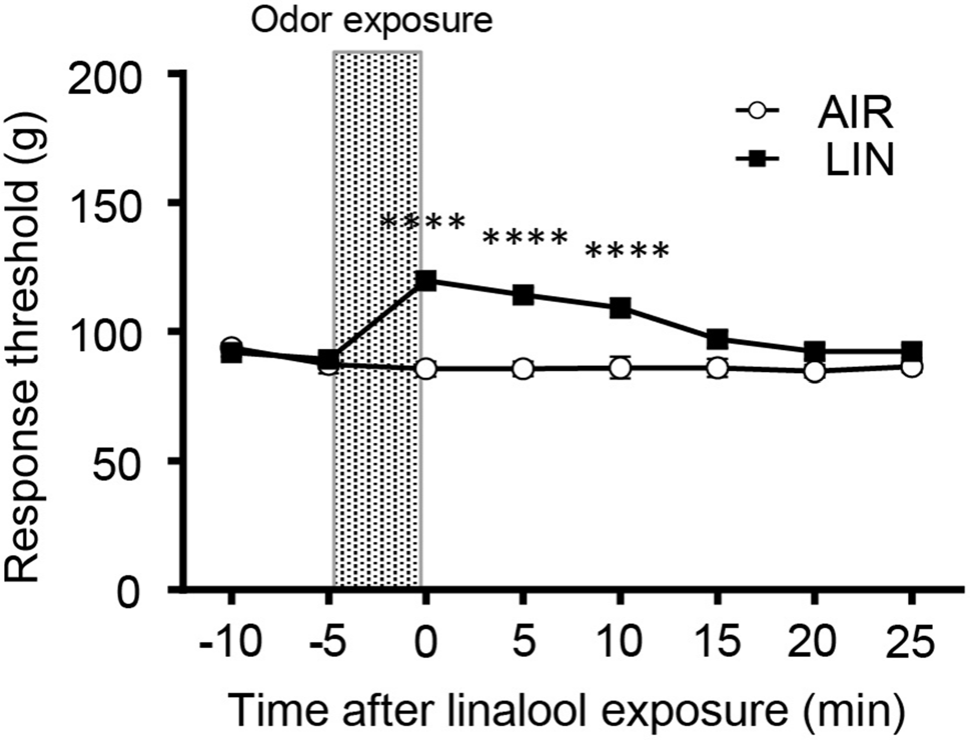
Five minutes of linalool odor exposure induced a sustained 10-min analgesic effect in the tail pincher test. The graph indicates alteration of the behavioral response threshold for mechanical nociceptive stimulation. The variation among animals was low enough that the error bars are hard to see in many time points. *n* = 10 for both odorless-air (AIR) exposed mice and linalool odor-exposed mice (LIN). The shaded area indicates odor exposure. Values are represented as mean ± SEM. ****p < 0.0001 (Sidak’s multiple comparison test).

Previously, we showed that linalool odor exposure during pain assays induced significant analgesic effects in mice^20^. In this study, we first examined the preventive effects of linalool odor on acute pain. Figure 1 shows the alteration of the mechanical nociceptive threshold as assessed on the tail pincher test. Two-way repeated measures ANOVA revealed that the main effects of odor treatment and time were significant (*F*_odor_[1, 18] = 32.88, *p* < 0.0001, *η*^2^ = 0.245; *F*_time_[7, 126] = 8.617, *p* < 0.0001, *η*^2^ = 0.138). The interaction between odor treatment and time was also significant (*F*_odor x time_[7, 126] = 12.09, *p* < 0.0001, *η*^2^ = 0.194). Sidak’s multiple comparison tests revealed that the threshold significantly increased with a large effect size (Cohen’s *d* > 0.8) for 10 min after linalool odor exposure (*p*_0min_ < 0.0001, *d* = 3.671, *p*_5min_ < 0.0001, *d* = 3.196, *p*_10min_ < 0.0001, *d* = 1.826, Supplementary Table 1) after linalool odor exposure.

**Figure 2:**
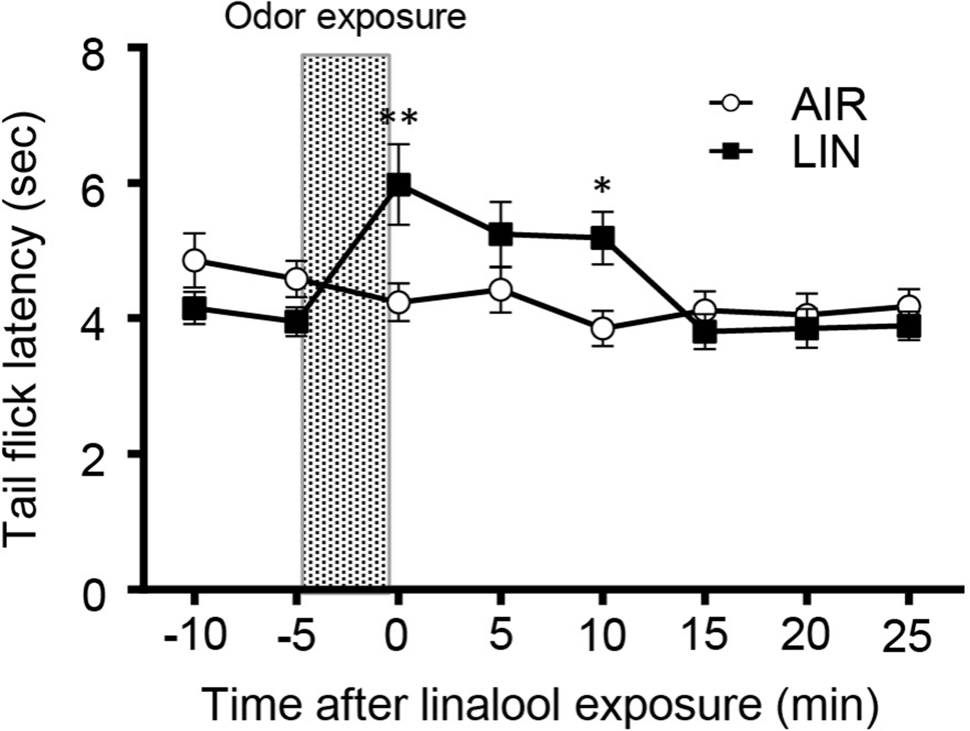
Five minutes of linalool odor exposure induced a sustained 10-min analgesic effect in the tail immersion test. The graph indicates the alteration of tail flick latency for thermal nociceptive stimulation. *n* = 10 for both odorless-air exposed mice (AIR) and linalool odor-exposed mice (LIN). The shaded area indicates odor exposure. Values are represented as mean ± SEM. **p < 0.005; *p < 0.05 (Sidak’s multiple comparison test).

The analgesic effects were also observed for thermal and chemical nociceptive stimulation. Figure 2 shows the alteration of tail flick latency that was assessed the thermal nociceptive threshold on the tail immersion test. Two way repeated measures ANOVA revealed that there are significant interaction between linalool odor exposure and time (*F*_odor x time_[7, 126] = 5.788, *p* < 0.0001, *η*^2^ = 0.143). Sidak’s multiple comparison test revealed that the response latency significantly increased for 10 min (*p*_0min_ = 0.0025, *d* = 1.181; *p*_5min_ = 0.509, *d* = 0.617, *p*_10min_ = 0.040, *d* = 1.273, Supplementary Table 2).

**Figure 3:**
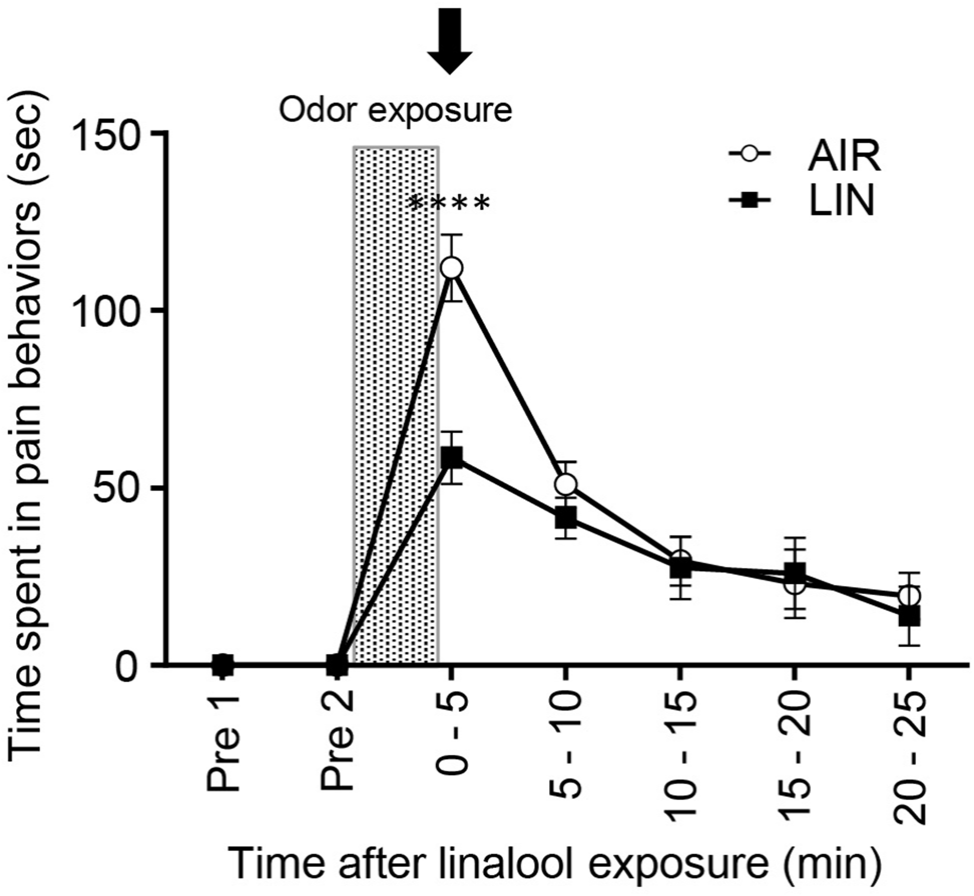
Five minutes of linalool odor exposure induced a sustained 5-min analgesic effect in the tail capsaicin test. Plots indicate the cumulative time spent in pain behaviors for every 5 min. *n* = 8 for both odorless-air exposed mice (AIR) and linalool-exposed mice (LIN). The shaded area indicates odor exposure. Arrow indicates the timing of capsaicin injection to the tail. Values are represented as mean ± SEM. ****p < 0.0001 (Sidak’s multiple comparison test).

Figure 3 shows the alteration of the time spent in pain behavior to chemical nociceptive stimulation as assessed on the tail capsaicin test. The time was significantly reduced in the linalool odor-exposed group (*F*_odor_[1, 14] = 4.308, *p* = 0.057, *η*^2^ = 0.0196, *F*_time_[6, 84] = 42.66, *p* < 0.0001, *η*^2^ = 0.637, *F*_odor x time_[6.84] = 4.700, *p* = 0.0004, *η*^2^ = 0.0702, two-way repeated measures ANOVA) during the first 5 min after linalool odor exposure (*p*_0-5 min_ < 0.0001, *d* = 2.235, Supplementary Table 3). We should note that pain behaviors in the capsaicin test rapidly decreased by 5 min after capsaicin injection; thus, it might be impossible to detect the effect of linalool odor exposure until 5 min after the injection.

Taken together, we concluded that 5 min prior exposure to linalool odor had a significant analgesic effect on the subsequent nociceptive stimulation.

### Orexin 1 receptors but not orexin 2 receptors in the spinal cord are essential for linalool odor-induced analgesia

**Figure 4:**
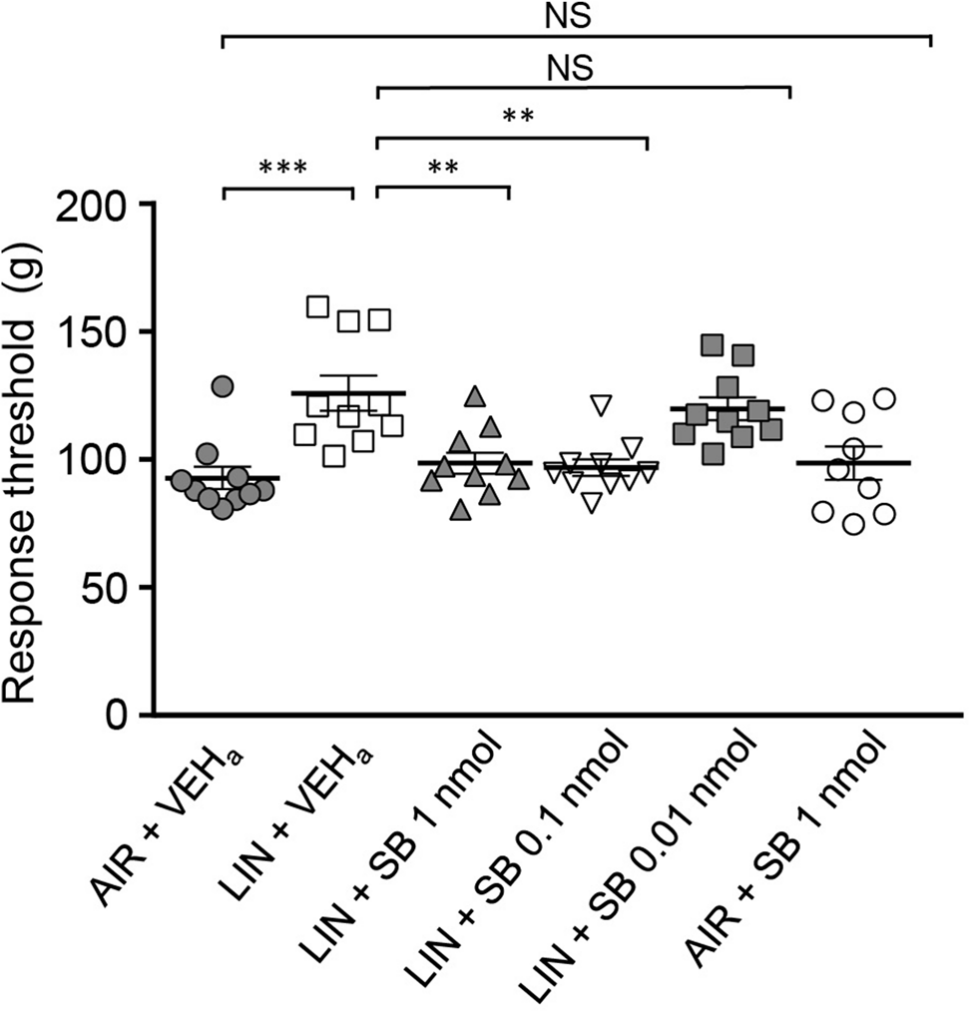
Intrathecal injection of SB 334867 suppressed linalool odor-induced analgesia in the tail pincher test. Each group consisted of 10 mice except for AIR + SB 1 nmol group (*n* = 9). AIR; odorless air exposed mice, LIN; linalool odor-exposed mice, VEH_a_; mice which received intrathecal injection of vehicle for antagonist, SB; mice which received intrathecal injection of OX1 receptor antagonist. Bars represent mean ± SEM. ***p < 0.0005; **p < 0.005 (Tukey’s multiple comparison test).

Linalool odor-induced analgesia in orexin-mutant mice revealed that orexinergic transmission is essential for analgesia^20^. We then examined the contribution of orexinergic transmission in the spinal cord by intrathecal injection of selective orexin receptor antagonists (SB 334867 for OX1 receptor, and TCS OX2 29 for OX2 receptor). Figure 4 shows that intrathecal injection of SB 334867 completely suppressed linalool odor-induced analgesia in tail pincher tests. Tukey’s multiple comparison test revealed that mechanical nociceptive threshold significantly increased after linalool odor exposure (*p* = 0.0003, *d* = 1.816, Supplementary Table 4), and the increase was prevented significantly by intrathecal injection of an OX1 receptor-selective antagonist in a dose-dependent manner (VEH vs 0.01 nmol SB 334867: *p* = 0.9532, *d* = 0.3353; VEH vs 0.1 nmol SB 334867: *p* = 0.0018, *d* = 1.712; VEH vs 1 nmol SB 334867: *p* = 0.0041, *d* = 1.521). The threshold almost returned to basal level at 0.1 nmol and 1 nmol (VEH vs 0.1 nmol SB 334867:*p* = 0.993, *d* = 0.330; VEH vs 1 nmol SB334867: *p* = 0.963, *d* = 0.431). Note that intrathecal injection of SB 334867 without linalool odor exposure did not show any significant effect.

**Figure 5:**
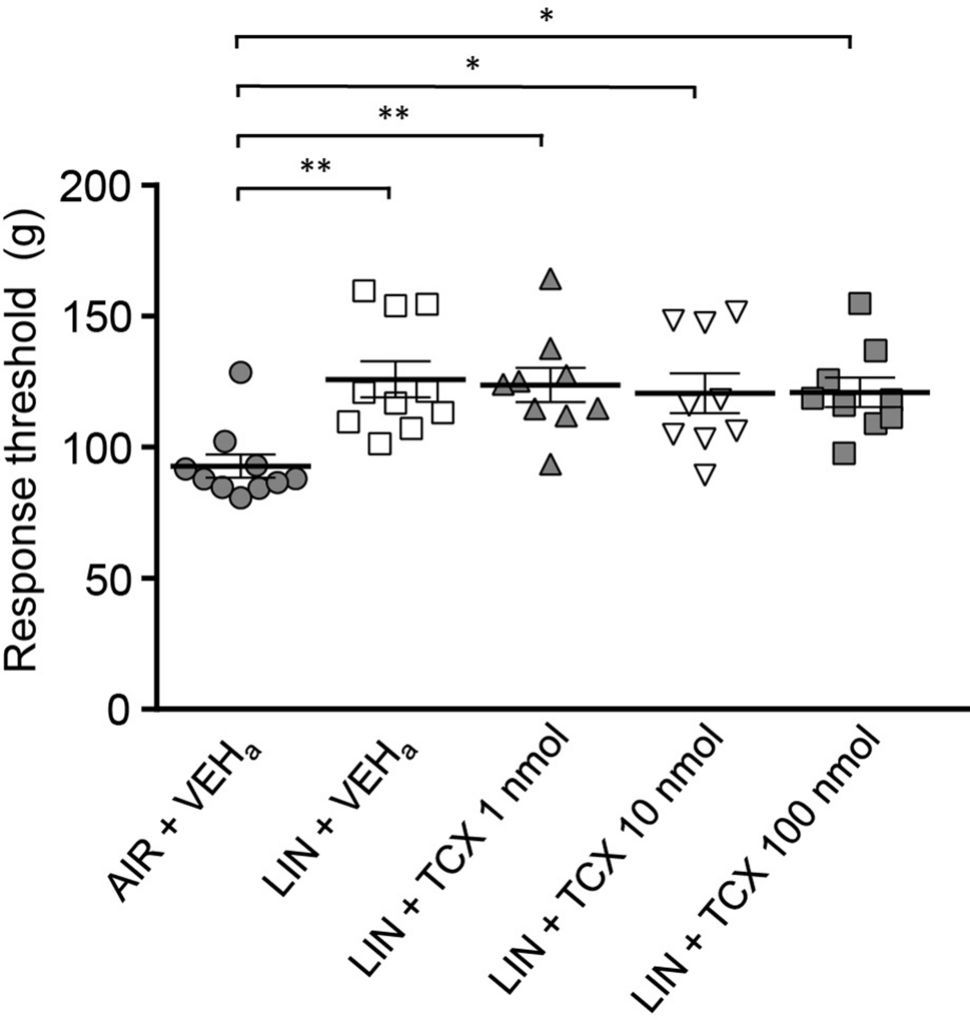
Intrathecal administration of TCX OX2 29 (orexin 2 receptor antagonist) did not suppress linalool odor-induced analgesia in the tail pincher test. *n* = 10 for AIR + VHE_a_ and LIN + VHE_a_ group. *n* = 9 for LIN + TCX 1 nmol, LIN + TCX 10 nmol, and LIN + TCX 100 nmol group. AIR; odorless air exposed mice, LIN; linalool odor-exposed mice, VEHa; mice which received intrathecal injection of vehicle for antagonist, TCS; mice which received intrathecal injection of OX2 receptor antagonist. Bars represent mean ± SEM. **p < 0.01, *p < 0.05 (Tukey’s multiple comparison test).

In contrast, the OX2 receptor antagonist did not show significant preventive effects (Fig. 5). The response threshold was not significantly altered after intrathecal injection of TCS OX2 29 with odor exposure, and the significant analgesic effect of linalool odor was maintained (VEH vs 1 nmol TCS OX2 29: *p* = 0.0091, *d* = 1.843; VEH vs 10 nmol TCS OX2 29: *p* = 0.024, *d* = 1.843; VEH vs 100 nmol TCS OX2 29: *p* = 0.022, *d* = 1.841, Supplementary Table 5).

Taken together, we found that orexinergic transmission in the spinal cord via OX1 receptor was essential for linalool odor-induced analgesia. Since the cell bodies of orexinergic neurons are localized in the hypothalamus and spinal cord receive direct axonal input from hypothalamic orexinergic neurons, our results indicate that the descending orexinergic pain inhibitory pathway is activated on linalool odor stimulation, which drives orexinergic synaptic transmission via OX1 receptor in the spinal cord.

### Intrathecal injection of SB 334867 did not prevent detection of linalool odor

Considering that orexinergic fibers are observed in the olfactory system^3,5,32^, it is possible that intrathecal injection of an OX1 receptor antagonist may interrupt the olfactory system and prevent detection of linalool odor. To examine this possibility, we assessed detection of linalool odor using an olfactory habituation/dishabituation test with intrathecal OX1 receptor antagonist administration.

**Figure 6:**
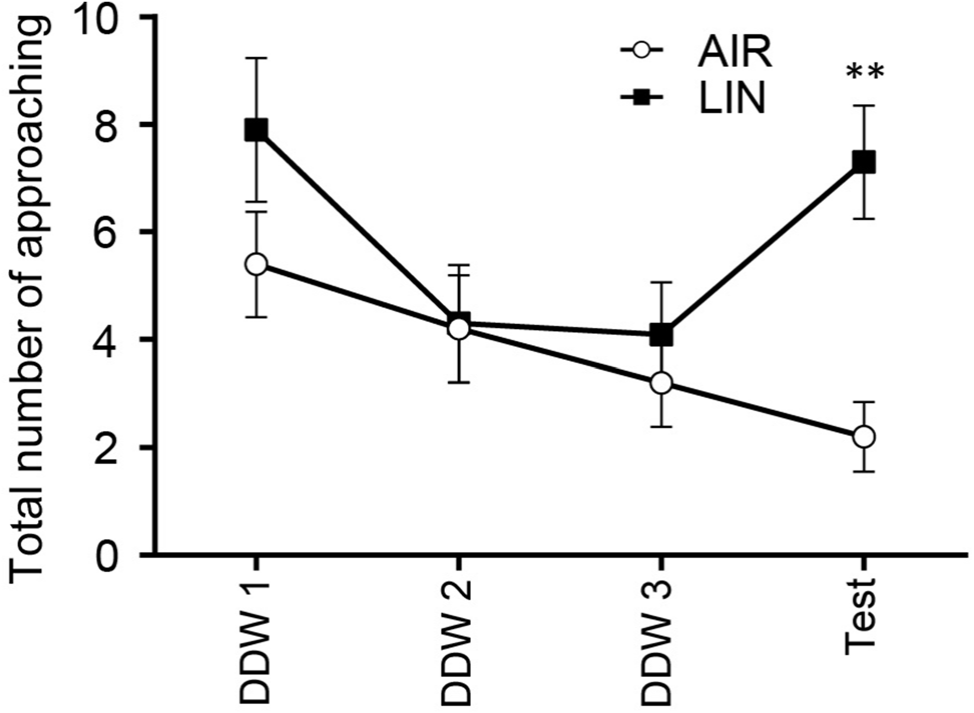
Olfactory habituation/dishabituation test confirmed the ability to detect the linalool odor under intrathecal administration of an orexin 1 receptor antagonist. Each dot represents the number of approaching to the cotton swab. DDW1, DDW2, DDW3: First, second, and third exposure to cotton swab soaked in 20 μL of double distilled water (DDW); Test: exposure to a swab soaked in 20 μL of linalool (linalool group) or with 20 μL of DDW (control group). *n* = 10 for both groups. Values are represented as mean ± SEM. **p < 0.005 between control and linalool odor-exposed group in the Test session (Sidak’s multiple comparison test).

Figure 6 shows the change in the number of approaches toward the cotton swab. Although the number gradually decreased by repeated presentation of a cotton swab soaked with DDW, the number increased when a swab soaked with linalool was presented at the test session. Two-way repeated measures ANOVA revealed that the time and the interaction between odor and time showed significant effects with large effect sizes (*F*_time_[3, 54] = 8.055, *p* = 0.0002, *η*^2^ = 0.1021; *F*_odor x time_[3, 54] = 5.820, *p* = 0.0016, *η*^2^ = 0.07372). Sidak’s multiple comparison test revealed that at the test session, the number of approaches to the cotton swab in the linalool-exposed group was significantly higher than that in the control DDW group (*p* = 0.0023, *d* = 1.844, Supplementary Table 6). These results indicate that intrathecal injection of SB 334867 did not prevent the detection of linalool odor.

### Intrathecal injection of SB 334867 alters activation of spinal cord neurons

**Figure 7:**
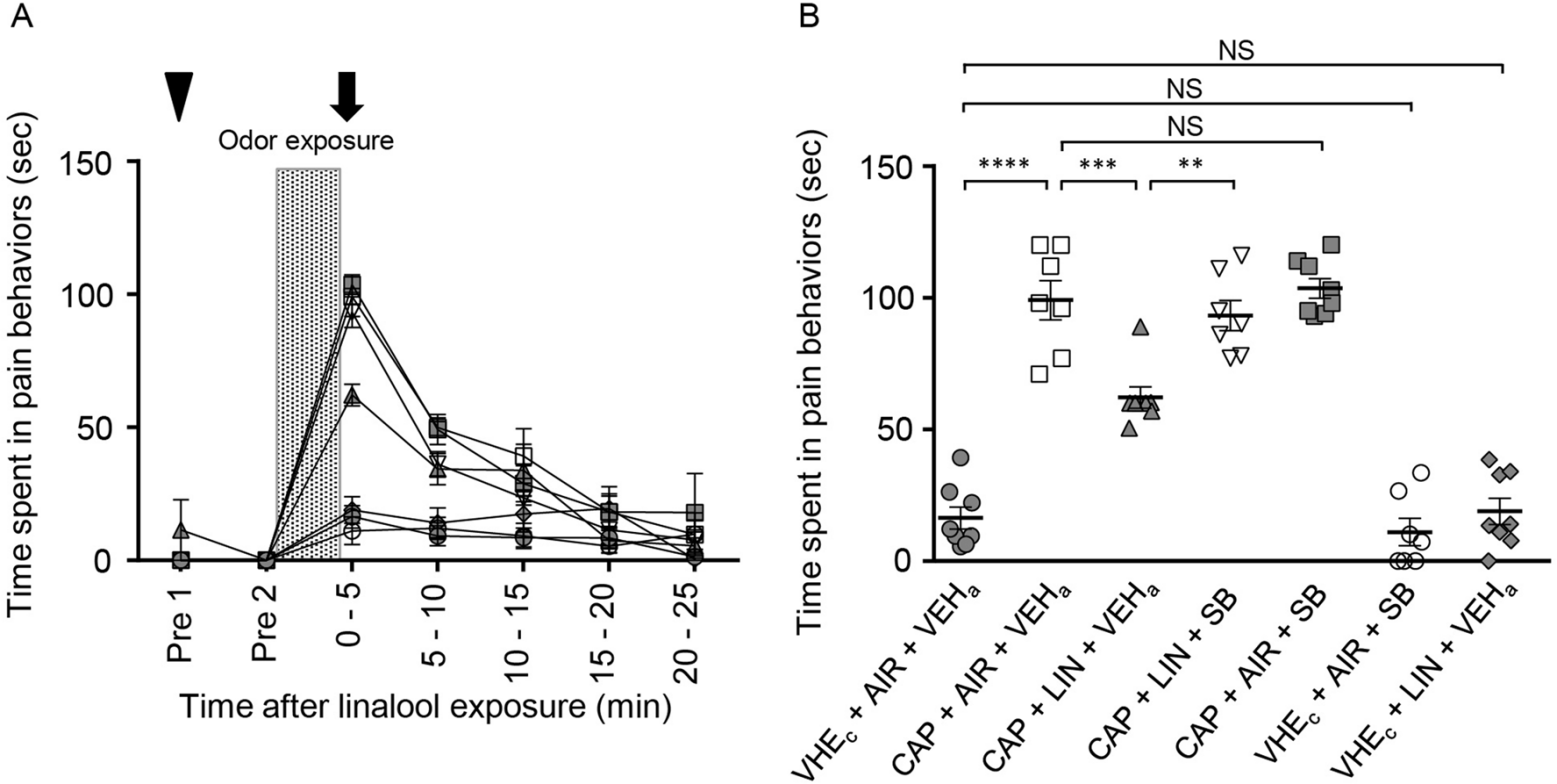
Suppression of capsaicin-induced pain behavior by linalool odor exposure was antagonized by intrathecal administration of orexin 1 receptor antagonist. (**A**) Values represent the time spent in pain behavior as mean ± SEM. The shaded area indicates odor exposure. Arrow and arrow head indicate the timing of intra-tail capsaicin injection and intrathecal SB-334867 injection. White square shows; CAP + AIR + VEH_a_, White inverted triangle shows; CAP + LIN + SB, White circle shows; VEH_c_ + AIR + SB. Gray circle shows; VHE_c_ + AIR + VEH_a_, Gray triangle shows; CAP + LIN + VEH_a_, Gray square shows; CAP + AIR + SB, Gray rhombus VHE_c_ + LIN + VEH_a_. (**B**) Time spent in pain behavior during the first 5 min after the capsaicin test are re-plotted. *n* = 7 for CAP + AIR + VEH_a_, CAP + LIN + SB, VEH_c_ + AIR + SB group, and *n* = 8 for VEH_c_ + AIR + VEH_a_, CAP + LIN + VEH_a_, CAP + AIR + SB, VEH_c_ + LIN + VEH_a_ group. AIR; odorless air exposed mice, LIN; linalool odor-exposed mice, VEH_c_; mice which received subcutaneous injection of vehicle for capsaicin, CAP; mice which received subcutaneous injection of capsaicin, VEH_a_; mice which received intrathecal injection of vehicle for antagonist, SB; mice which received intrathecal injection of 1 nmol OX1 receptor antagonist. Bars represent mean ± SEM. ****p < 0.0001, ***p < 0.0005, **p < 0.005 (Sidak’s multiple comparison test).

To assess the effect of intrathecal injection of an OX1 receptor antagonist on the activity of spinal cord neurons induced by peripheral noxious stimulation, we performed immunohistochemical analyses of c-Fos expression, a marker of neuronal activity in the spinal cord. In the preliminary experiments, we examined c-Fos expression induced by the tail pincher test, but we could not detect a significant increase in c-Fos positive cells in the spinal cord after the tail pincher test (data not shown). We further examined it after the tail capsaicin test (Fig. 7). Because the analgesic effect of linalool odor was evident during the first 5 min after linalool odor exposure (Fig. 3, Fig. 7A), we performed the statistical analyses at this time point (Fig. 7B). Tukey’s multiple comparison test revealed that the time spent in pain behavior significantly increased after capsaicin injection in the tail (*p* < 0.0001, *d* = 5.168, Supplementary Table 7), but this increase was prevented by prior linalool odor exposure (*p* = 0.0001, *d* = 2.340). The preventive effect was antagonized by intrathecal injection of 1 nmol SB 334867 (*p* = 0.0015, *d* = 2.340). Note that intrathecal injection of SB 334867 without linalool odor exposure did not show a significant effect (*p* = 0.990, *d* = 0.413). In addition, linalool odor exposure itself did not induce a significant change in the pain behavior without peripheral noxious stimulus (*p* = 0.998, *d* = 0.413).

**Figure 8:**
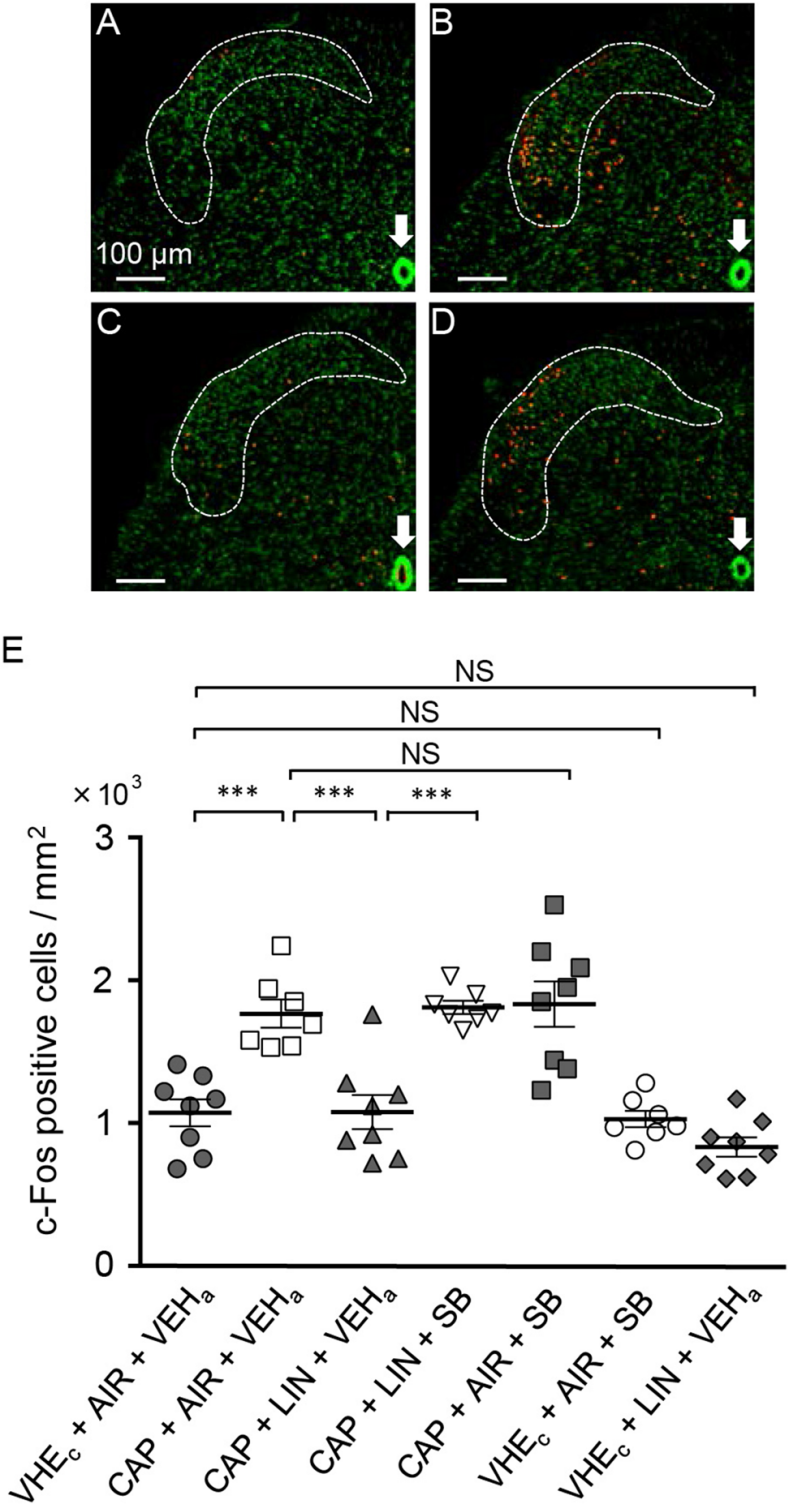
Suppression of capsaicin-induced c-Fos expression in the spinal cord by linalool odor exposure was antagonized by intrathecal orexin 1 receptor antagonist administration. (**A**–**D**) c-Fos expression in laminae 1 and 2 of the S1 segment (dashed line) are represented at the condition of VEH_c_ + AIR + VEH_a_ (**A**), CAP + AIR + VEH_a_ (**B**), CAP + LIN + VEH_a_ (**C**), and CAP + LIN + SB (**D**). Scale bars in (**A**–**D**) indicate 100 μm. Arrows in (**A**–**D**) indicate the Central Canal. (**E**) Densities of c-Fos positive cells in laminae 1 and 2 of the S1 segment after the tail capsaicin test were plotted. AIR; odorless air exposed mice, LIN; linalool odor-exposed mice, VEH_c_; mice which received subcutaneous injection of vehicle for capsaicin, CAP; mice which received subcutaneous injection of capsaicin, VEH_a_; mice which received intrathecal injection of vehicle for antagonist, SB; mice which received intrathecal injection of 1 nmol OX1 receptor antagonist. Bars represent mean ± SEM. *** *p* < 0.0005 (Sidak’s multiple comparison test).

We next examined the expression of c-Fos in the dorsal horn of the S1 segment after capsaicin injection in the tail and assessed the effect of intrathecal injection of an OX1 receptor antagonist. The number of c-Fos-positive cells increased in response to capsaicin injection in the tail (Fig. 8A,B); however, this increase was prevented by linalool odor exposure (Fig. 8C). The prevention was significantly antagonized by intrathecal injection of SB 334867 (Fig. 8D). Tukey’s multiple comparison test revealed that the density of c-Fos positive cells in the upper laminae (lamina 1 and 2) of the S1 dorsal horn significantly increased after capsaicin injection in the tail (*p* = 0.0004, *d* = 2.625, Supplementary Table 8), and this increase was prevented by linalool odor exposure (*p* = 0.0004, *d* = 2.239). Moreover, the prevention was significantly antagonized by intrathecal SB 334867 injection (*p* = 0.0002, *d* = 2.766), and the density almost returned to the level of capsaicin injection without linalool odor (*p* > 0.999, *d* = 0.210) (Fig. 8E). These results indicate that activation of spinal dorsal horn neurons evoked by peripheral chemical nociceptive stimulation (capsaicin injection) was prevented by linalool odor exposure via OX1 receptor. We should note that intrathecal injection of SB 334867 itself did not significantly change the density of c-Fos-positive cells (*p* > 0.999, *d* = 0.198). Furthermore, linalool odor exposure itself did not show a significant change in density (*p* = 0.624, *d* = 1.021) (Fig. 8E).

## Discussion

In this study, we showed that intrathecal administration of an OX1 receptor selective antagonist prevented the analgesic effects of linalool odor exposure. Since the cell bodies of orexinergic neurons are localized in the hypothalamus and spinal cord receive direct axonal input from the hypothalamic orexin neurons, our results indicate that the descending orexinergic pain inhibitory pathway is activated by linalool odor stimulation and drives synaptic transmission via OX1 receptor in the spinal cord.

### Technical limitations

In this study, we showed that orexinergic descending inhibition via OX1 receptor in the spinal cord was essential for linalool odor-induced analgesia in male mice. To have a general understanding of these phenomena, we need to further assess this effect on female mice to examine potential sex differences^33,34^. In addition, we investigated the descending orexinergic inhibition driven by linalool odor exposure in the acute pain model. To reveal the effect of orexinergic inhibition on chronic pain, further studies are required^27,35,36^.

### Preventive effects of linalool odor-induced analgesia

Previously, we showed that linalool odor exposure during the pain test induced a significant analgesic effect^20^. However, the preventive effect of linalool odor-induced analgesia has not been addressed yet. In this study, the behavioral threshold for mechanical nociception significantly increased after linalool odor exposure, and it was maintained for at least 10 min (Fig. 1), indicating that a short linalool odor exposure induced a sustained analgesic effect. The preventive analgesic effect was observed with noxious heat (Fig. 2) and chemical (Fig. 3) stimulation, indicating that odor-induced analgesia prevents multimodal nociceptive input. The neuronal mechanisms underlying the preventive effect are yet to be elucidated. Previous studies have indicated that synaptic transmission from olfactory sensory neurons to olfactory bulb neurons ceases a few seconds after the end of odor stimulation^37,38^, suggesting that the neuronal circuit mechanisms in the central olfactory system or higher centers of the brain might contribute to the sustained (~ 10 min) effect. A subpopulation of mitral/tufted cells, which are projection neurons of the main olfactory bulb, showed persistent spike discharges evoked by short odor stimulation^39,40^. A persistent discharge of mitral/tufted cells might contribute to the sustained analgesic effect of the linalool odor.

### Orexinergic descending inhibitory pathway in odor-induced analgesia

Orexin peptide-deficient mice and orexin neuron-ablated mice did not show linalool odor-induced analgesic effect; therefore, orexinergic synaptic transmission plays a pivotal role in odor-induced analgesia^20^. However, the neuronal circuits responsible for the analgesia have not yet been addressed. In this study, we focused on the spinal cord, the first relay of the central pain pathway. Immunohistochemical studies have indicated that orexin-containing fibers were present, but orexin-containing cell bodies were not observed in the spinal cord^30,31^, and the cell bodies of orexin neurons were highly localized in the lateral hypothalamus^1,3–5^, indicating that orexin neurons have direct axonal input to the spinal cord. Since intrathecal injection of orexin peptide induced significant analgesic effects^16,27,28,35,41^, the orexinergic input to the spinal cord could act as one of the descending inhibitory pathways^42^.

In this study, we showed that the increase of the threshold of mechanical pain behaviors induced by linalool odor exposure was largely (> 80%) antagonized by intrathecal OX1 receptor antagonist administration (Fig. 4). Furthermore, the decrease of chemical pain behaviors was also largely (> 80%) antagonized by the OX1 receptor antagonist (Fig. 7). These results indicate that the orexinergic pain inhibitory pathway mediated by OX1 receptor in spinal cord is the major route for linalool odor-induced analgesia. But we should also note that less than 20% of the analgesic effects induced by linalool odor exposure were remained after the intrathecal administration of OX1 receptor antagonist, indicating the minor but substantial contribution of other analgesic circuit(s). Previous studies have revealed that the orexinergic transmission at supraspinal level could contribute to the pain processing^19,22–26^. Consequently, the supraspinal orexinergic system could also play a role for linalool odor-induced analgesia.

In this study, we examined the contribution of orexinergic descending inhibition to the analgesic effect of linalool odor. In addition to the olfactory sensory route, systemic^43–46^ or intrathecal^47^ administration of linalool also mediate the analgesic effects. Though the involvement of adenosine A1 and A2a receptors for systemic administration-induced analgesic effect^45^, the involvement of orexinergic system for the analgesic effects of linalool via systemic rout has not yet examined and further analysis is required to address the point.

### Neuronal circuit mechanisms in the spinal cord underlying the odor-induced analgesia

Gene expression and protein distribution analyses revealed that OX1 receptor is expressed in dorsal horn neurons^21,48,49^. Electrophysiological studies have indicated that orexin regulates synaptic transmission via OX1 receptor^50,51^. Furthermore, intrathecal administration of an OX1 receptor antagonist suppressed the OX1 receptor-induced analgesic effects^16,28,41,52,53^. These results suggest that the orexinergic descending inhibitory pathway may suppress pain transmission pathway in the spinal cord dorsal horn via OX1 receptor.

In contrast to the effect of an OX1 receptor antagonist, intrathecal administration of an OX2 receptor antagonist did not antagonize linalool odor-induced analgesia (Fig. 5). Immunohistochemical analysis has revealed that OX2 receptor is localized in lamina 2 of the spinal dorsal horn^54^. Moreover, an electrophysiological study has reported that a subset of lamina 2 neurons of the spinal cord is activated via OX2 receptor^55^ Taken together, there may be subsets of the orexinergic descending inhibition pathway, among which only a subset was activated on linalool odor stimulation.

Activation of OX1 receptor increased intracellular Ca^2+^ concentration and induced cell excitation^30,56–58^ therefore, the involvement of inhibitory neurons in the spinal cord may be essential for orexinergic suppression of pain information processing. Consistent with this hypothesis, an electrophysiological study has shown that orexin-A evoked inhibitory synaptic input via OX1 receptor of lamina 2 neurons of the spinal dorsal horn^51^.

Immunohistochemical mapping of c-Fos revealed that capsaicin-induced activation of dorsal horn neurons was suppressed by linalool odor exposure. Furthermore, the suppression was antagonized by intrathecal administration of OX1 receptor antagonist (Fig. 8). These results are consistent with the hypothesis that linalool odor exposure activates hypothalamic orexinergic neurons and the descending orexinergic input drives the inhibitory interneurons and suppresses excitatory synaptic transmission in the spinal dorsal horn. We also noted that linalool odor exposure without noxious stimuli did not increase the number of c-Fos-positive cells in lamina 2 of the spinal dorsal horn (Fig. 8E), suggesting that the orexinergic input suppressed the noxious-stimuli-evoked activity without activating the cell body of inhibitory interneurons. This implies that the orexinergic axon may terminate on the presynaptic axon terminals of the inhibitory interneurons and modulate the release of inhibitory synaptic transmitter via OX1 receptors expressed on the presynaptic site^56^.

## Conclusion

We found that orexinergic descending inhibition was essential for linalool odor-induced analgesia in mice. Furthermore, orexinergic synaptic transmission via OX1 receptor in the spinal cord plays a pivotal role in this effect. These findings not only provide a foothold to elucidate the neuronal circuit mechanisms underlying odor-induced analgesia but also a potential therapeutic approach for treating pain with orexinergic descending inhibition.

## Methods

### Animals

Wild-type mice (C57BL/6J, 21–30 g, n = 273, CLEA Japan Inc., Tokyo, Japan) were used. All experiments were performed on male mice to avoid possible differences associated with estrous cycling in females. The animals were maintained with lights on at 7. a.m. and off at 7:00 p.m. at a constant temperature (23 ± 1 °C). All experiments were performed during the light cycle, between 12:00 p.m. and 6:00 p.m. To avoid the carry-over effect, each animal was used only once to test for linalool odor, medication, and pain. Animals were handled and acclimatized to experimental equipment for 6 days. On the experiment day, mice were transferred to the laboratory 3 h before the start of the experiment. All experiments were performed according to the guidelines outlined by the Physiological Society of Japan and approved by the Laboratory Animal Research Committee of Kagoshima University.

### Drugs

Linalool (CAS# 78-70-6, > 96.0%, Tokyo Chemical Industry, Tokyo, Japan), capsaicin ((E)-N-(4-Hydroxy-3-methoxyphenyl) methyl)-8-methyl-6-nonenamide, CAS# 404-86-4, > 95%, Sigma-Aldrich Tokyo, Tokyo, Japan), SB 334867 (N-(2-methyl-6-benzoxazolyl)-N′-1,5-naphthyridin-4-yl urea, CAS# 792173-99-0, > 99%, Tocris Bioscience, Bristol, UK), TCS OX2 29 ((2*S*)-1-(3,4-Dihydro-6,7-dimethoxy-2(1*H*)-isoquinolinyl)-3,3-dimethyl-2-[(4-pyridinylmethyl) amino]-1-butanone hydrochloride, CAS# 1610882-30-8, > 98%, Tocris Bioscience, Bristol, UK) were used. Capsaicin was dissolved in a vehicle containing 10% ethanol (CAS# 64-17-5, > 94.8–95.8%, Nacalai Tesuque, Kyoto, Japan)/10% Tween 80 (polyoxyethylene sorbitan monooleate, CAS# 9005-65-6, Wako, Tokyo, Japan)/80% saline. SB 334867 and TCS OX 29 were dissolved using the following methods. First, SB334867 was dissolved in DMSO (dimethyl sulfoxide, CAS# 67-68-5, > 99%, Wako, Tokyo, Japan) to a concentration of 50 mM. Next, the solution was diluted to the required concentration (0.002 mM, 0.02 mM, 0.2 mM for SB 384467; 0.2 mM, 2 mM, 20 mM for TCS OX 29) using 45% β-cyclodextrin ((2-Hydroxypropyl)-β-cyclodextrin, CAS# 128446-35-5, > 97%, Sigma-Aldrich Tokyo, Tokyo, Japan) in artificial cerebrospinal fluid (ACSF). We used 0.4% DMSO/45% β-CD in ACSF as the vehicle control for intrathecal administration. We confirmed that the vehicle did not cause any apparent behavioral abnormality in our preliminary experiment^59^.

### Acclimatization

Before measuring the nociceptive threshold, mice were allowed to become familiar with the experimenter who handled them for 5 min, gently wrapped them in a cotton towel for 5 min, and by gently touching the tail with the fingers several times. Then, the mouse was placed in an observation chamber (top diameter: 8 cm, base diameter: 11.5 cm, height: 15 cm, content: 1 L) for 5 min. In the capsaicin test, a mouse was additionally acclimatized for 3 min with a handmade animal holder (a 50 mL Falcon tube cut to a length of 8 cm with a hole for free breathing). The mice were then acclimatized to the observation chamber. The acclimatization session was performed once per day and repeated for at least 6 days till the mice showed no escape behavior during the acclimatization session. In every behavioral experiment, each mouse was acclimatized to the laboratory for 3 h before the start of the experiment.

### Linalool odor exposure

Odor exposure was performed using a custom-made olfactometer^20^. Briefly, 0.5 mL of linalool dispensed into a glass vial (content: 20 mL) was vaporized in an odor chamber (content: 0.32 L) at room temperature (23 ± 1 °C). Clean air deodorized with a charcoal filter and double distilled water (DDW) was introduced into the odor chamber from a compressed air cylinder, and the output odorized air was ventilated into the observation chamber (content: 1 L) at a constant rate (1 L/min). After 10 min of pre-ventilation of odorized air, a mouse was individually placed in the observation chamber and exposed to the odorous air for 5 min. Immediately after the odor exposure, we performed the pain assay to examine the nociceptive behavior. Since the humidity of the carrier gas and the temperature of the odor chamber were kept constant, the concentration of the odorous gas was considered constant. When a mouse was exposed to odorless air, a blank glass vial was placed in the odor chamber. All observation chambers were changed between each trial and were cleaned up to prevent the effect of lingering scents.

### Tail pincher test

To assess the mechanical pain threshold, we performed the tail pincher test with calibrated forceps (BIO-RPM, Bioseb, Vitrolles, France)^60,61^. Before the test session, we marked the position 1/3rd of the distance from the tip of the tail. After 5 min of odor exposure, the mouse was immediately and gently restrained with a towel and pressure was applied on the marking on the tail with calibrated forceps. We recorded the latency when the mouse flicked, withdrew its tail, or struggled in the cotton towel. We measured the threshold 5 times repeatedly (trial interval: 10–15 s). Subsequently, the maximum and minimum values of the 5 trials were discarded, and the average value from the remaining 3 trials was considered as the value for that time point^61^. To prevent the mouse from being injured, a cut-off pressure point was set at 500 g. Each animal was used only once to prevent hyperalgesia.

### Tail immersion test

To assess the thermal pain threshold, we performed the tail immersion test^62^. Before the test session, we marked the position 2/3rd of the distance from the tip of the tail with a felt pen. Five minutes after odor exposure, the mice were gently restrained with a towel and the tail was soaked till the mark in a circulating water bath heated to 47 °C. The latency of the mouse flicking his tail or struggling was recorded. We measured the threshold at 5 min intervals. To prevent the mouse from being injured, the cut-off time was set at 20 s.

### Tail capsaicin test

To assess the chemical pain response, we performed the tail capsaicin test modified from the classical foot capsaicin test^63,64^ and the tail formalin test^65^. Immediately after 5 min of odor exposure, the mouse was placed in a restraining device. Twenty microliters of 0.3% capsaicin were injected intradermally into the dorsal surface of the tail using a Hamilton syringe with a 30-gauge needle within 1 min^65^. The mice were returned to their individual cages, and the cumulative duration of pain behavior was measured every 5 min for up to 25 min using a stopwatch to measure the time spent in pain behaviors (shaking and licking of the tail).

### Intrathecal drug administration

To assess the effect of orexin receptor antagonists on odor-induced analgesia in the spinal cord, we performed acute intrathecal administration of drugs 10 min before odor exposure. An unanesthetized mouse was gently wrapped in a cotton towel, and the needle insertion site was disinfected with 70% ethanol. A disposable 30-gauge ½-in. needle attached to a 10-μL Hamilton microsyringe was inserted into the L5–L6 intervertebral space^66^. We observed sudden twitching of the tail as a sign of dural penetration^67^. The volume of intrathecal drug administration was 5 μL. During our visual inspection, none of the mice showed any walking dysfunction. We completed intrathecal administration within 5 min to reduce stress.

### Olfactory habituation/dishabituation test

To assess whether mice could detect the linalool odor after intrathecal drug administration, we performed olfactory habituation/dishabituation tests^68–70^. Ten minutes after the intrathecal drug injection, the mouse was placed in an acrylic cage (12 cm × 20 cm × 10 cm) with a wire-mesh lid and was first exposed to a cotton swab soaked with 20 μL of DDW three times for 2 min (habituation trials) and then to a cotton swab soaked with 20 μL of linalool for 2 min (dishabituation test trial). Control mice were exposed to a swab soaked with 20 μL of DDW for 2 min in the dishabituation test trial. In this behavioral paradigm, repetitive exposure to the cotton swab soaked with DDW (habituation trials) causes the mouse to rapidly lose the interest to the cotton swab and the investigating behaviors to the swab are rapidly decreased. But in case that the mouse is exposed to the cotton swab soaked with an odorous solution after the habituation trials, the mouse recognizes the odorized swab as the novel one and re-investigates it (dishabituation test trial). The number of times they approached the cotton swab was recorded as exploratory behavior. Approach was defined as the action of the mouse moving its nose within 10 mm of the cotton swab.

### Immunohistochemistry

Ninety minutes after the tail capsaicin test, the mice were deeply anesthetized with urethane (1.3 g/kg, i.p.) and transcardially perfused with saline followed by 4% paraformaldehyde in 0.01 M PBS (pH 7.4). The spinal cord was then excised, post-fixed at 4 °C for 3 h, and cryoprotected with 30% sucrose in 0.01 M PBS. Thereafter, the first segment of the sacral spinal cord (S1) was carefully cut out because nociceptive fibers which innervate tail make synaptic input in the S1^71^, and 30-μm sections were made with a cryostat (Microtome Cryostat HM500, Thermo Fisher scientific, MA, USA). Every alternate section was collected, and floating immunohistochemical staining was performed. Sections were incubated with PBS containing 0.3% Triton-X and 1% normal horse serum for 30 min, then allowed to react with rabbit anti c-Fos monoclonal antibody (9F6, 1/1000, Cell Signaling Technology, Danvers, MA, USA) overnight at 4 °C. After rinsing with PBS, the sections were incubated with secondary antibodies (CF568-conjugated anti-rabbit IgG, 1/400, Biotium, Heyward, CA, USA) for 90 min in a dark box at room temperature. Sections were then rinsed with PBS and stained with NeuroTrace 640/660 deep-red (1/100, Thermo Fisher Scientific, Waltham, MA, USA) for fluorescent Nissle staining to identify lamina 2 of the dorsal horn of the spinal cord^72^. The sections were mounted on a glass slide and examined under a fluorescence microscope (BZ-X700, KEYENCE, Osaka, Japan). We counted the number of c-Fos-positive cells in the upper lamina (laminae 1–2) of all stained sections and represented as the density of c-Fos positive cells (/mm^2^) in the upper lamina of the spinal cord. We determined the number of c-Fos positive cells for each mouse by calculating the mean of the three sections possessing the most c-Fos-positive cells from all sections of S1 spinal cord^73,74^.

### Statistical analyses

If not otherwise specified, statistical comparisons were performed using one-way ANOVA with post-hoc Tukey’s multiple comparison tests or two-way repeated measures ANOVA with post-hoc Sidak’s multiple comparison tests using Prism7 software (GraphPad Software, San Diego, CA, USA.). The criterion for statistical significance was *p* < 0.05 in all cases. We also calculated Cohen’s *d* (for comparison of two groups) or η^2^ (for ANOVA) as the effect size. The criteria for large effect size was *d* > 0.8, *η*^2^ > 0.14; medium, *d* > 0.5, *η*^2^ > 0.06; small, *d* > 0.2, *η*^2^ > 0.01^75^. All statistical values for multiple comparisons were tabulated in Supplemental Information, and only essential values were represented in the result section.

### Methods statement

All methods were carried out in compliance with local safety regulations and applicable ARRIVE guidelines.

## Supplementary Information

Supplementary Information.

## Data Availability

The datasets generated during and/or analyzed during the current study are available from the corresponding author on reasonable request.
