# Supplementary material for: Orexinergic descending inhibitory pathway mediates linalool odor-induced analgesia in mice
Source: Sci Rep. 2021 Apr 29;11:9224. doi: 10.1038/s41598-021-88359-5 (PMC8085205; doi:10.1038/s41598-021-88359-5)
Supplement: Supplementary file 1 — Supplementary Information. [file 41598_2021_88359_MOESM1_ESM.pdf]

Orexinergic descending inhibitory pathway mediates linalool odor-induced analgesia in mice

Yurina Higa[1,2,3], Hideki Kashiwadani[1,3,*], Mitsutaka Sugimura[2], Tomoyuki Kuwaki[1]

[1]Departments of Physiology, Graduate School of Medical and Dental Sciences, Kagoshima University, Kagoshima 890-8544, Japan

[2]Department of Dental Anesthesiology, Graduate School of Medical and Dental Sciences, Kagoshima University, Kagoshima 890-8544, Japan

[3]These authors equally contributed to this study

*Corresponding author

Supplementary Table 1. Summary of response threshold, significance level, and effect size on odor-induced analgesia in tail pincher test

			time after odor exposure							
Groups	sample size		-10 min	-5 min	0 min	5 min	10 min	15 min	20 min	25 min
AIR	10	response threshold (g)	93.72 ± 3.739	87.21 ± 3.465	85.52 ± 2.942	85.52 ± 2.942	85.99 ± 4.298	85.90 ± 3.487	84.63 ± 2.412	86.36 ± 2.093
LIN	10	response threshold (g)	91.97 ± 1.707	89.31 ± 2.467	119.8 ± 2.962	114.32 ± 2.828	109.2 ± 3.721	97.02 ± 3.212	92.18 ± 2.869	92.25 ± 2.432
		significance level	p = 0.9987	p = 0.9987	p < 0.0001	p < 0.0001	p = 0.0033	p = 0.1159	p = 0.689	p = 0.4358
		effect size	d = 0.1911	d = 0.2208	d = 3.671	d = 3.196	d = 1.8257	d = 1.048	d = 0.9008	d = 0.8206

Response thresholds are represented as mean ± SEM. p values are adjusted by Tukey's method. Effect sizes indicate Cohen's d. AIR: odorless air exposed group; LIN: linalool odor exposed group

Supplementary Table 2. Summary of response latency, significance level, and effect size on odor-induced analgesia in tail immersion test

Groups	sample size		time after odor exposure							
			-10 min	-5 min	0 min	5 min	10 min	15 min	20 min	25 min
AIR	10	response latency (sec)	4.856 ± 0.399	4.584 ± 0.266	4.238 ± 0.278	4.424 ± 0.340	3.849 ± 0.258	4.119 ± 0.277	4.048 ± 0.3	4.179 ± 0.249
LIN	10	response latency (sec)	4.152 ± 0.236	3.950 ± 0.216	1.887 ± 0.597	5.24 ± 0.484	5.187 ± 0.393	3.802 ± 0.261	3.851 ± 0.2	3.887 ± 0.212
		significance level	p = 0.6915	p = 0.7953	p = 0.0025	p = 0.5085	p = 0.0401	p = 0.9962	p = 0.9999	p = 0.9978
		effect size	d = 0.679	d = 0.827	d = 1.181	d = 0.617	d = 1.273	d = 0.372	d = 0.207	d = 0.400

Response latencies are represented as mean ± SEM. p values are adjusted by Tukey's method. Effect sizes indicate Cohen's d. AIR: odorless air exposed group; LIN: linalool odor exposed group

Supplementary Table 3. Summary of duration of pain responses, significance level, and effect size on odor-induced analgesia in tail capsaicin test

Groups	sample size		time after odor exposure						
			pre1	pre2	0-5 min	5-10 min	10-15 min	15-20 min	20-25 min
AIR	8	response latency (sec)	0 ± 0	0 ± 0	112.0 ± 9.487	51.00 ± 6.372	29.38 ± 6.897	23.00 ± 9.646	19.50 ± 6.609
LIN	8	response latency (sec)	0 ± 0	0 ± 0	58.50 ± 7.297	41.5 ± 5.754	27.5 ± 8.793	25.88 ± 10.09	13.88 ± 8.350
		significance level	-	-	p <0.0001	p = 0.9270	p > 0.9999	p = 0.9997	p = 0.9924
		effect size	-	-	d = 2.235	d = 0.553	d = 0.084	d = 0.103	d = 0.264

Cumurative pain behavior of 5 min are represented as mean ± SEM. p values are adjusted by Sidak's method. Effect sizes indicate Cohen's d. Because there were no pain behaivors before capsaicin injection, the significane level and effect size were not calcurated at the time points. AIR: odorless air exposed group; LIN: linalool odor exposed group

Supplementary Table 4-1. Summary of response threshold and sample size for the effect of OX1 receptor antagonist on tail pincher test

	SB_1+AIR	VEH_a+AIR	VEH_a+LIN	$SB_{0.01}$+LIN	$SB_{0.1}$+LIN	SB_1+LIN
sample size	9	10	10	10	10	10
response threshold	98.63 ± 6.537	92.817 ± 4.386	126.0 ± 6.881	119.8 ± 4.414	96.84 ± 3.237	98.63 ± 4.145

Supplementary Table 4-2. Summary of significane level and effect size for multiple comparison of the effect of OX1 receptor antagonist.

	SB_1+AIR	vehicle+AIR	VEH_a+LIN	$SB_{0.01}$+LIN	$SB_{0.1}$+LIN	SB_1+LIN
SB_1+AIR	-	p = 0.9667<br>d = 0.3451	p = 0.0055<br>d = 1.3157	p = 0.056<br>d = 1.256	p = 0.9999<br>d = 0.1159	p > 0.9999<br>d < 0.0001
VEH_a+AIR		-	p = 0.0003<br>d = 1.816	p = 0.0046<br>d = 1.941	p = 0.9927<br>d = 0.3300	p = 0.9626<br>d = 0.4306
VEH_a+LIN			-	p = 0.9532<br>d = 0.3353	p = 0.0018<br>d = 1.712	p = 0.0041<br>d = 1.521
$SB_{0.01}$+LIN				-	p = 0.0238<br>d = 1.878	p = 0.0459<br>d = 1.566
$SB_{0.1}$+LIN					-	p = 0.9999<br>d = 0.1519
SB_1+LIN						-

Response thresholds are represented as mean ± SEM. p values were adjusted by Tukey's method. d values are effect sizes calculated by Cohen's method. SB_1:1nmol SB334867 administered group, $SB_{0.1}$: 0.1nmol SB334867 administered group, $SB_{0.01}$: 0.01nmol SB334867 administered group, VEH_a: vehicle administered group, AIR: odorless air exposed group, LIN: linalool odor exposed group

Supplementary Table 5-1. Summary of response threshold and sample size for the effect of OX2 receptor antagonist on tail pincher test

	VEH_a+AIR	VEH_a+LIN	TCS_1+LIN	TCS_{10}+LIN	TCS_{100}+LIN
sample size	10	10	9	9	9
response threshold	92.82 ± 4.386	126.0 ± 6.881	123.8 ± 6.531	120.7 ± 7.653	121.0 ± 5.573

Supplementary Table 5-2. Summary of significane level and effect size for multiple comparison of the effect of OX2 receptor antagonist.

	VEH_a+AIR	VEH_a+LIN	TCS_1+LIN	TCS_{10}+LIN	TCS_{100}+LIN
VEH_a+AIR	-	p = 0.0034<br>d = 1.816	p = 0.0091<br>d = 1.843	p = 0.0236<br>d = 1.486	p = 0.0216<br>d = 1.841
VEH_a+LIN		-	p = 0.9992<br>d = 0.103	p = 0.9745<br>d = 0.237	p = 0.9794<br>d = 0.249
TCS_1+LIN			-	p = 0.9967<br>d = 0.148	p = 0.9978<br>d = 0.157
TCS_{10}+LIN				-	p > 0.9999<br>d = 0.015
TCS_{100}+LIN					-

Response thresholds are represented as mean ± SEM. p values were adjusted by Tukey's method. d values are effect sizes calculated by Cohen's method. TCS_1:1nmol TCS OX 29 administered group, TCS_{10}: 10nmol TCS OX 29 administered group, TCS_{100}: 100nmol TCS OX 29 administered group, VEH_a: vehicle administered group, AIR: odorless air exposed group, LIN: linalool odor exposed group

Supplementary Table 6. Summary of response threshold, significance level, and effect size on odor-induced analgesia in tail pincher test

Groups	sample size		session			
			DDW1	DDW2	DDW3	TEST
CON	10	investigation time (sec)	5.400 ± 0.980	4.200 ± 0.998	3.200 ± 0.814	2.200 ± 0.646
LIN	10	investigation time (sec)	7.900 ± 1.337	4.300 ± 1.086	4.100 ± 0.960	7.300 ± 1.055
		significance level	p = 0.4323	p > 0.9999	p = 0.929	p = 0.0036
		effect size	d = 0.6745	d = 0.0303	d = 0.3199	d = 1.844

Investigating times are represented as mean ± SEM. p values are adjusted by Sidak's method. Effect sizes indicate Cohen's d. CON: DDW exposed group at test session; LIN: linalool exposed group at test session.

Supplementary Table 7-1. Summary of duration of pain behavir and sample size for the effect of OX1 receptor antagonist on tail capsaicine test (0-5 min)

	VEH_c+AIR+VEH_a	CAP+AIR+VEH_a	CAP+LIN+VEH_a	CAP+LIN+SB	CAP+AIR+SB	VEH_c+AIR+SB	VEH_c+LIN+VEH_a
sample size	8	7	8	7	8	7	8
duration of pain behavior (sec)	16.38 ± 4.193	99.17 ± 7.457	62.13 ± 4.016	93.29 ± 5.764	103.6 ± 3.690	11.10 ± 5.195	18.98 ± 5.007

Supplementary Table 7-2. Summary of significance level and effect size for the effect of OX1 receptor antagonist on tail capsaicine test (0-5 min)

	VEH_c+AIR+VEH_a	CAP+AIR+VEH_a	CAP+LIN+VEH_a	CAP+LIN+SB	CAP+AIR+SB	VEH_c+AIR+SB	VEH_c+LIN+VEH_a
VEHc+AIR+VEHa	-	p < 0.0001<br>d = 5.168	p < 0.0001<br>d = 3.941	p < 0.0001<br>d = 5.677	p < 0.0001<br>d = 7.812	p = 0.9898<br>d = 0.4133	p = 0.9998<br>d = 0.1994
CAP+AIR+VEHa		-	p = 0.0001<br>d = 2.340	p = 0.9848<br>d = 0.3335	p = 0.9958<br>d = 0.2891	p <0.0001<br>d = 5.180	p < 0.0001<br>d = 4.196
CAP+LIN+VEHa			-	p = 0.0015<br>d = 2.340	p < 0.0001<br>d = 3.806	p < 0.0001<br>d = 4.073	p < 0.0001<br>d = 3.361
CAP+LIN+SB				-	p = 0.779<br>d = 0.802	p < 0.0001<br>d = 5.662	p < 0.0001<br>d = 5.061
CAP+AIR+SB					-	p < 0.0001<br>d = 7.652	p < 0.0001<br>d = 6.805
VEHc+AIR+SB						-	p = 0.9267<br>d = 0.5644
VEHc+LIN+VEHa							-

Duration of pain behaviors are represented as mean ± SEM. p values were adjusted by Tukey's method. d values are effect sizes calculated by Cohen's method. VEH_a: mice received intrathecal administration of vehicle for SB334867, VEH_c:mice received intradermal administration of vehicle for capsaicin, AIR: odorless air exposed group, LIN: linalool odor exposed group, CAP: capsaicin administered group, SB: 1 nmol SB334867 administered group,

Supplementary Table 8-1. Summary of c-Fos(+) cell density and sample size for the effect of OX1 receptor antagonist on tail capsaicine test

	VEH_c+AIR+VEH_a	CAP+AIR+VEH_a	CAP+LIN+VEH_a	CAP+LIN+SB	CAP+AIR+SB	VEH_c+AIR+SB	VEH_c+LIN+VEH_a
sample size	8	7	8	7	8	7	8
c-Fos(+) cell density ($x10^3/mm^2$)	1.073 ± 0.0946	1.767 ± 0.0987	1.079 ± 0.1212	1.810 ± 0.0471	1.834 ± 0.1595	1.029 ± 0.0583	0.8338 ± 0.0686

Supplementary Table 8-2. Summary of significance level and effect size for the effect of OX1 receptor antagonist on tail capsaicine test

	VEH_c+AIR+VEH_a	CAP+AIR+VEH_a	CAP+LIN+VEH_a	CAP+LIN+SB	CAP+AIR+SB	VEH_c+AIR+SB	VEH_c+LIN+VEH_a
VEHc+AIR+VEHa	-	p = 0.0004<br>d = 2.625	p > 0.9999<br>d = 0.0203	p = 0.0001<br>d = 3.459	p < 0.0001<br>d = 2.053	p > 0.9999<br>d = 0.1978	p = 0.6236<br>d = 1.021
CAP+AIR+VEHa		-	p = 0.0004<br>d = 2.239	p > 0.9999<br>d = 0.2100	p = 0.9992<br>d = 0.1778	p = 0.0002<br>d = 3.444	p < 0.0001<br>d = 4.096
CAP+LIN+VEHa			-	p = 0.0002<br>d = 2.766	p < 0.0001<br>d = 1.885	p = 0.9999<br>d = 0.185	p = 0.5950<br>d = 0.880
CAP+LIN+SB				-	p > 0.9999<br>d = 0.070	p < 0.0001<br>d = 5.574	p < 0.0001<br>d = 5.905
CAP+AIR+SB					-	p < 0.0001<br>d = 2.328	p < 0.0001<br>d = 2.880
VEHc+AIR+SB						-	p = 0.8318<br>d = 1.103
VEHc+LIN+VEHa							-

Duration of pain behaviors are represented as mean ± SEM. p values were adjusted by Tukey's method. d values are effect sizes calculated by Cohen's method. VEH_a: mice received intrathecal administration of vehicle for SB334867, VEH_c:mice received intradermal administration of vehicle for capsaicin, AIR: odorless air exposed group, LIN: linalool odor exposed group, CAP: capsaicin administered group, SB: 1 nmol SB334867 administered group,
